# Preoperative global longitudinal strain predicts myocardial hypertrophy burden but not the magnitude of reverse remodeling after surgical aortic valve replacement in severe aortic stenosis

**DOI:** 10.1186/s44348-026-00089-2

**Published:** 2026-09-04

**Authors:** Joy Wulansari Purba, Ario Soeryo Kuncoro, Rina Ariani, Estu Rudiktyo, Amiliana Mardiani Soesanto

**Affiliations:** 1 Department of Cardiovascular, Adam Malik Hospital, Medan, Indonesia; 2 Department of Cardiology and Vascular Disease, Faculty of Medicine, Universitas Sumatera Utara, Medan, Indonesia; 3 Department of Cardiology and Vascular Medicine, Faculty of Medicine, Universitas Indonesia. National Cardiovascular Center Harapan Kita, Jakarta, Indonesia

**Keywords:** Aortic valve stenosis, Global longitudinal strain, Ventricular remodelling, Surgical aortic valve replacement, Echocardiography

## Abstract

**Background:**

Global longitudinal strain (GLS) is a marker of subclinical myocardial dysfunction and predicts adverse outcomes in severe aortic stenosis. However, its ability to predict the magnitude of left ventricular (LV) reverse remodeling following surgical aortic valve replacement (SAVR) remains uncertain.

**Methods:**

In 94 patients with severe aortic stenosis and preserved ejection fraction undergoing SAVR, patients were stratified by baseline GLS (normal, < –16%; abnormal, ≥ –16%). Given incomplete follow-up, the principal analysis was a complete-case comparison of interventricular septal dimension (IVSd), LV mass index (LVMI), relative wall thickness, and ejection fraction across baseline, post-SAVR, and follow-up in 54 patients with complete data, supplemented by correlation analyses treating baseline GLS as a continuous variable. A mixed-effects model in all 94 patients was a secondary analysis.

**Results:**

In the complete-case cohort, the abnormal GLS group had greater IVSd and LVMI and lower ejection fraction at baseline, with these differences persisting at each time point. A significant GLS-by-time interaction was observed for LVMI (P** = **0.005). Continuous GLS correlated with absolute LVMI and IVSd at all time points but not with the magnitude of change in any index. After adjustment for baseline disease severity, continuous GLS was not independently associated with any remodeling outcome (all P ≥ 0.121). Baseline GLS identified a greater myocardial hypertrophy burden but does not predict the magnitude of reverse remodeling after SAVR.

**Conclusions:**

Preoperative GLS reflects the severity of preexisting LV hypertrophy but, with the possible exception of LV mass regression, does not independently predict the magnitude of early reverse remodeling after SAVR once baseline disease severity is accounted for.

**Supplementary Information:**

The online version contains supplementary material available at https://doi.org/10.1186/s44348-026-00089-2.

## Background

Aortic stenosis (AS) is the most common degenerative valve disease in developed countries, characterized by progressive obstruction of the left ventricular (LV) outflow tract, leading to chronically increased afterload [1]. In response, the LV undergoes concentric hypertrophy to normalize wall stress, a process that is initially adaptive but in the long-term can lead to diastolic dysfunction, myocardial fibrosis, and eventually heart failure [2]. Aortic valve replacement (AVR), either surgical AVR (SAVR) or transcatheter AVR, is the only definitive therapy that effectively eliminates obstruction, triggering a recovery process known as reverse remodeling [3]. This process, characterized by the regression of LV mass (LVM) and functional improvement, is a critical determinant of long-term clinical outcomes after intervention [4].

In recent years, global longitudinal strain (GLS), measured by speckle-tracking echocardiography, has emerged as a more sensitive parameter than LV ejection fraction (LVEF) for detecting subclinical myocardial dysfunction [5]. In patients with AS and preserved LVEF, a reduction in GLS is often an early marker of intrinsic myocyte damage and interstitial fibrosis [6]. Consequently, preoperative GLS has been established as a potent predictor of adverse clinical outcomes, including mortality, following AVR [7, 8]. However, uncertainty remains regarding the role of preoperative GLS in predicting the magnitude or capacity of structural reverse remodeling. Intuitively, it might be assumed that a myocardium with worse subclinical function (abnormal GLS) would have a more limited recovery capacity. In contrast, some studies suggest that baseline structural parameters, such as LVM, are the primary predictors of mass regression, consistent with the law of initial values [9]. Understanding this relationship is crucial for risk stratification, managing expectations, and potentially optimizing the timing of intervention in patients with severe AS.

Therefore, this study aimed to evaluate the relationship between preoperative GLS, both as a dichotomous category and as a continuous variable, and the magnitude of reverse remodeling at three time points: pre-SAVR, post-SAVR, and at follow-up (after discharge), using a complete-case analytic framework as the principal approach.

## Methods

### Ethics statement

The study was approved by The Committee on Institutional Review Board/Health Research Ethics of National Cardiac Center Harapan Kita Hospital(No. DP.04.03/KEP092/EC049/2025). Informed consent was obtained in accordance with the Declaration of Helsinki.

### Study design and study population

This was a retrospective, observational cohort study involving patients with symptomatic severe AS and preserved ejection fraction (EF) who underwent SAVR. Data were obtained from the patient database of the National Cardiovascular Center Harapan Kita (NCCHK) of Indonesia. A total of 94 patients who met the inclusion criteria and had the necessary echocardiographic data were included in the final analysis. The exclusion criteria were significant concomitant mitral valvular disease, congenital disease, previous cardiac surgery, and inadequate echocardiographic image quality.

#### Echocardiographic data collection

Comprehensive transthoracic echocardiography data were acquired at three time points: before SAVR (baseline), during in-hospital evaluation after SAVR, and at follow-up (after discharge). All measurements were performed in accordance with the guidelines of the American Society of Echocardiography (ASE) and the European Association of Cardiovascular Imaging [10].

End-diastolic interventricular septal dimension (IVSd) was measured using the parasternal long-axis view. LV GLS was measured from the apical four-, two-, and three-chamber views using speckle-tracking software (ViewPoint 6, EchoPAC; GE Healthcare). The average GLS value of the 17 segments was used for the analysis. Preserved EF was defined as EF > 50%. LV internal diameter (LVIDd), IVSd, and posterior wall thickness (PWd) were measured at end-diastole using 2D-guided M-mode imaging. LV mass was calculated using the ASE-corrected cube formula and indexed to body surface area to obtain the LVM index (LVMI). Relative wall thickness (RWT) was calculated as 2 × PWd/LVIDd.

LVEF was measured using the biplane modified Simpson method from apical two- and four-chamber views. LV end-diastolic volume (LVEDV) and LV end-systolic volume (LVESV) were traced at end-diastole and end-systole, and LVEF was computed as (LVEDV − LVESV)/LVEDV × 100. All measurements were performed offline by an experienced echocardiographer blinded to the patients' clinical data.

#### Variable definitions

The primary predictor variable was baseline GLS category, defined based on a cutoff of –16%, which was identified as the optimal value based on normative thresholds [11, 12]. Patients were classified into two groups: normal GLS (< –16%, better myocardial function) and abnormal GLS (≥ –16%, subclinical myocardial dysfunction). As GLS quantifies the percentage of myocardial shortening, more negative values reflect better-preserved myocardial function. The outcome variables were IVSd thickness (mm), LVMI (g/m^2^), RWT, and LVEF (%), all measured at the three time points.

#### Statistical analysis

Statistical analyses were performed using IBM SPSS ver. 30.0 (IBM Corp). Descriptive statistics were presented as mean** ± **standard deviation for continuous variables and frequencies (percentages) for categorical variables. Between-group comparisons were performed using a two-sided Welch t-test or, for non-normally distributed variables (assessed by the Shapiro–Wilk test), using the Mann–Whitney U-test. We used a linear mixed-effects model (LMM) to analyze longitudinal changes in reverse-remodeling parameters (IVSd, LVMI, RWT, and EF). The LMM was selected because it can account for repeated measurements within the same participant and accommodate missing data under the missing-at-random (MAR) assumption.

Given that 42.6% of patients lacked echocardiographic data at the mid-term follow-up time point, this degree of missingness was considered excessive for reliable trend estimation using a mixed-effects framework alone, even under the MAR assumption. We therefore adopted a complete-case analysis, restricted to the 54 patients with echocardiographic data available at all three time points (baseline, early post-SAVR, and follow-up), as the principal analytic approach. The pattern of missingness at follow-up was examined by comparing baseline and post-AVR characteristics between patients with and without follow-up data using independent t-tests, and by correlating each baseline/post-AVR variable with follow-up status.

For each outcome (IVSd, LVMI, RWT, EF), values were compared between GLS groups at each of the three time points, and within-group change over time (pre-AVR to post-AVR, and pre-AVR to follow-up) was tested. A GLS × time interaction term was used to test whether the magnitude of change over time differed between GLS groups. These analyses were performed both in the complete-case cohort (primary, n = 54) and in the full cohort using all available pairwise data (secondary, *n* = 94).

To characterize the relationship between baseline GLS as a continuous variable and the magnitude of reverse remodeling, Spearman correlation coefficients were calculated between baseline GLS and the absolute value of each outcome after AVR and at follow-up, and between GLS and the magnitude of change (Δ) in each outcome from baseline to post-AVR and from baseline to follow-up. Multivariable linear regression was then used to test whether continuous baseline GLS was independently associated with the change in each outcome after adjustment for baseline disease severity. Model 1 was adjusted for the baseline value of the respective outcome, peak transvalvular gradient, mean transvalvular gradient, and aortic valve area (AVA). Model 2 was additionally adjusted for sex, smoking status, and tricuspid annular plane systolic excursion (TAPSE).

As a secondary, exploratory analysis, the LMM was used to analyze longitudinal trajectories using all available data (*n* = 94) under the MAR assumption. The association between the pattern of missing data at follow-up and the baseline GLS category was examined using a chi-square test; as no significant association was found (*P* = 0.946), the MAR assumption was considered plausible for this exploratory model. Separate LMMs were constructed for each outcome, with GLS category and time included as fixed effects, an interaction term for GLS × time, and subject ID included as a random intercept. Because this model relies on the MAR assumption in the setting of substantial missingness, its results are presented as supportive rather than primary evidence.

Echocardiographic timing intervals (baseline to SAVR, SAVR to post-SAVR, SAVR to follow-up, and baseline to follow-up) were compared between GLS groups using a two-sided Welch independent-samples t-test to evaluate whether systematic differences in assessment timing could confound the remodeling trajectories. A two-sided *P*-value of < 0.05 was considered statistically significant throughout.

## Results

### Baseline characteristics and missing data pattern

A total of 94 patients with severe AS who underwent SAVR were included in the final analysis of the study. Based on a prespecified cutoff of –16%, 72 patients (76.6%) were classified into the abnormal GLS group (GLS ≥ –16%), and 22 patients (23.4%) were classified into the normal GLS group (GLS < –16%). The baseline characteristics of the study population stratified by GLS category are presented in Table 1.

**Table 1:** Baseline characteristics of the study population (*n* = 94)

Characteristic	Normal GLS (*n* = 22)	Abnormal GLS (*n* = 72)	*P*-value
Male sex	5 (22.70)	49 (68.10)	< 0.001^*^
Age (yr)	60.50 ± 9	60 ± 12	0.795
Body mass index (kg/m^2^)	24.52 ± 4.21	24.53 ± 4.19	0.990
Hypertension	9 (40.90)	32 (44.40)	0.770
Diabetes mellitus	1 (4.50)	15 (20.80)	0.106
Chronic kidney disease	0 (0)	1 (1.40)	> 0.999
Smoker	3 (13.60)	31 (43.10)	0.012^*^
Indication to CABG	3 (13.60)	14 (19.40)	0.754
Indication to TV repair ring annuloplasty	0 (0)	7 (9.70)	0.194
Hospital stay (day)	7.50 ± 2	8 ± 8	0.078
Echocardiographic parameter			
LVEF (%)	65.00 ± 7.61	60.50 ± 7.48	0.017^*^
IVSd (mm)	12.06 ± 2.67	14.40 ± 3.16	0.024^*^
RWT	0.52 ± 0.15	0.56 (0.19)	0.091
LVMI (g/m^2^)	111.50 ± 102.25	164.00 ± 66.25	0.011^*^
LVIDd (mm)	47.85 ± 8.20	48.14 ± 7.36	0.514
LAVI (mL/m^2^)	38.50 ± 7.75	45.76 ± 16.17	0.153
TAPSE (mm)	24.81 ± 3.68	21.62 ± 5.40	0.011^*^
Peak pressure gradient (mmHg)	85.72 ± 29.83	107.03 ± 32.25	0.007^*^
Mean pressure gradient (mmHg)	57.36 ± 17.29	68.04 ± 22.01	0.040^*^
Pre-AVR GLS (%)	–18.68 ± 2.11	–11.60 ± 4.55	< 0.001^*^
AVA (cm^2^)	0.69 ± 0.15	0.50 ± 0.23	< 0.001^*^

Values are presented as number (%) or mean ± standard deviation

AVA, aortic valve area; AVR, aortic valve replacement; CABG, coronary artery bypass grafting; GLS, global longitudinal strain; IVSd, interventricular septal dimension; LAVI, left atrial volume index; LVIDd, left ventricular internal dimension diastole; LVEF, left ventricular ejection fraction; LVMI, left ventricular mass index; RWT, relative wall thickness; TAPSE, tricuspid annular plane systolic excursion; TV, tricuspid valve

^*^P < 0.05

Table 1 presents the echocardiographic characteristics of the study population before AVR. Patients with abnormal GLS (≥ –16%) demonstrated significantly lower LVEF than those with normal GLS (60.50%** ± **7.48% vs. 65.00%** ± **7.61%, P** = **0.017). Indices of LV remodeling were also more pronounced in the abnormal GLS group, as shown by increased IVSd (14.40** ± **3.16 mm vs. 12.06** ± **2.67 mm, P** = **0.024) and higher LVMI (164.00 ± 66.25 g/m^2^ vs. 111.50** ± **102.25 g/m^2^, P** = **0.011). Although the RWT tended to be higher in this group, the difference was not statistically significant (P** = **0.091).

Furthermore, TAPSE was significantly lower in the abnormal GLS group (21.62 ± 5.40 mm vs. 24.81** ± **3.68 mm, P** = **0.011), indicating reduced right ventricular systolic function. Hemodynamically, patients with abnormal GLS showed higher peak and mean transaortic gradients (peak pressure gradient: 107.03** ± **32.25 mmHg vs. 85.72** ± **29.83 mmHg, P** = **0.007; mean pressure gradient: 68.04** ± **22.01 mmHg vs. 57.36** ± **17.29 mmHg, P** = **0.040) and a smaller AVA (0.50** ± **0.23 cm^2^ vs. 0.69** ± **0.15 cm^2^, P < 0.001).

Longitudinal IVSd data were available for all 94 patients at baseline and in-hospital post-AVR evaluation. At follow-up, data were missing for 40 patients (42.6%), yielding 54 patients (57.4%) with complete datasets at all three time points. None of the available baseline or post-AVR echocardiographic variables, including continuous GLS, IVSd, LVMI, RWT, and EF, differed significantly between patients with and without follow-up data (all P > 0.05), and the baseline GLS category was not associated with missingness (χ^2^ = 0.005, P = 0.946) (Tables 2, 3). The 40 patients without mid-term follow-up data were predominantly those unable to attend scheduled follow-up echocardiographic evaluations because they resided outside the study region, at a considerable distance from the study center. This pattern is consistent with the data being missing completely at random or MAR with respect to the measured echocardiographic variables, supporting both the complete-case analysis as the principal approach and the secondary LMM as a reasonable exploratory check.

**Table 2:** Comparison of baseline characteristics between patients with and without follow-up echocardiography

Characteristic	Follow-up echocardiography		*P*-value
	Yes (n = 54)	No (n = 40)	
GLS category			0.946
Abnormal	42 (77.8)	30 (75.0)	
Normal	12 (22.2)	10 (25.0)	
Baseline			
GLS (%)	− 13.13 ± 3.93	− 12.96 ± 4.50	0.843
IVSd (mm)	13.65 ± 2.65	14.13 ± 3.84	0.745
LVMI (g/m^2^)	162.37 ± 65.18	162.39 ± 57.36	0.655
RWT	0.56 ± 0.12	0.60 ± 0.18	0.182
EF (%)	61.04 ± 7.16	62.25 ± 8.44	0.343
SAVR-to-post-echocardiography interval (day)	7.07 ± 5.07	5.92 ± 4.72	0.226
Post-AVR			
IVSd (mm)	12.93 ± 2.60	13.23 ± 3.48	0.633
LVMI (g/m^2^)	131.20 ± 37.20	132.79 ± 49.80	0.860
EF (%)	58.72 ± 8.41	62.38 ± 12.08	0.062

Values are presented as mean ± standard deviation or number (%)

AVR, aortic valve replacement; EF, ejection fraction; GLS, global longitudinal strain; IVSd, interventricular septal dimension; LVMI, left ventricular mass index; RWT, relative wall thickness; SAVR, surgical aortic valve replacement

**Table 3:** Univariate predictors of follow-up missingness

Predictor	r	*P*-value
Baseline		
GLS (%)	+ 0.021	0.843
IVSd (mm)	+ 0.075	0.475
LVMI (g/m^2^)	< 0.001	0.999
EF (%)	+ 0.078	0.454
SAVR-to-post-echocardiography interval (day)	− 0.116	0.266
Post-AVR		
IVSd (mm)	+ 0.050	0.633
EF (%)	+ 0.177	0.087

AVR, aortic valve replacement; EF, ejection fraction; GLS, global longitudinal strain; IVSd, interventricular septal dimension; LVMI, left ventricular mass index; SAVR, surgical aortic valve replacement

### Echocardiographic timing between GLS groups

To rule out systematic timing differences as a confounder of the remodeling trajectories, we compared all echocardiographic timing intervals between GLS groups (Table S1). None of the four intervals (baseline to SAVR, SAVR to post-SAVR, SAVR to follow-up, and baseline to follow-up) differed significantly between the normal GLS and abnormal GLS groups (all P ≥ 0.122), indicating that the observed remodeling trajectories were not confounded by differential assessment timing.

We further tested whether variability in the timing of post-SAVR and follow-up assessments was itself associated with the magnitude of structural change (Table 4). Timing was not associated with ΔIVSd, ΔLVMI, or ΔRWT in either phase. A significant association was observed only between the SAVR-to-post-SAVR interval and ΔEF in phase 1 (ρ = − 0.261, P = 0.011), most plausibly reflecting acute perioperative hemodynamic variation rather than differences in long-term remodeling; this finding does not alter the overall interpretation of the study.

**Table 4:** Association between assessment timing and magnitude of remodeling (Δ)

Outcome (Δ)	Spearman ρ	*P*-value
Phase 1 (n = 94)		
Δ IVSd	− 0.158	0.128
Δ LVMI	− 0.040	0.705
Δ RWT	+ 0.041	0.694
Δ EF	− 0.261	0.011^*^
Phase 2 (n = 54)		
Δ IVSd	− 0.243	0.076
Δ LVMI	− 0.117	0.400
Δ RWT	− 0.038	0.785
Δ EF	− 0.012	0.930

Phase 1, SAVR-to-post-SAVR echocardiography interval. Phase 2, SAVR-to-follow-up echocardiography interval

EF, ejection fraction; GLS, global longitudinal strain; IVSd, interventricular septal dimension; LVMI, left ventricular mass index; RWT, relative wall thickness; SAVR, surgical aortic valve replacement

^*^P < 0.05

### Primary analysis: complete-case comparison of reverse remodeling

The primary analysis was conducted using a complete-case approach, restricted to participants who had echocardiographic data available at all three time points (baseline, early post-SAVR, and follow-up; n = 54). This approach was adopted to ensure reliable trend estimation given the 42.6% missingness at the follow-up time point. Results are summarized in Table 5.

**Table 5:** Complete-case comparison of reverse remodeling parameters by GLS group (n = 54)

Parameter	Normal GLS (n = 12)	Abnormal GLS (n = 42)	*P*-value
IVSd (mm)			
Pre-AVR	14.14 ± 2.51	11.94 ± 2.50	0.010^*^
Post-AVR	13.39 ± 2.56	11.28 ± 2.12	0.012^*^
Follow-up	11.97 ± 2.02	10.68 ± 2.42	0.053
Interval change			
Pre-AVR – post-AVR	–0.66 ± 1.79	–0.74 ± 2.33	0.787
Pre-AVR – follow-up	–1.27 ± 1.21	–2.16 ± 2.86	0.208
LVMI (g/m^2^)			
Pre-AVR	177.30 ± 65.00	110.20 ± 29.80	< 0.001^*^
Post-AVR	140.00 ± 35.70	100.50 ± 24.10	< 0.001^*^
Follow-up	118.00 ± 30.60	94.40 ± 29.20	0.015^*^
Interval change			
Pre-AVR – post-AVR	–9.80 ± 37.30	–37.30 ± 51.00	0.030^*^
Pre-AVR – follow-up	–15.80 ± 32.20	–59.30 ± 60.90	0.005^*^
RWT			
Pre-AVR	0.56 ± 0.11	0.52 ± 0.14	0.212
Post-AVR	0.62 ± 0.15	0.51 ± 0.12	0.006^*^
Follow-up	0.54 ± 0.17	0.53 ± 0.10	> 0.999
Interval change			
Pre-AVR – post-AVR	–0.01 ± 0.17	0.06 ± 0.17	0.246
Pre-AVR – follow-up	0.01 ± 0.18	–0.02 ± 0.21	0.588
EF (%)			
Pre-AVR	59.83 ± 7.13	65.25 ± 5.71	0.013^*^
Post-AVR	57.45 ± 8.78	63.17 ± 5.06	0.036^*^
Follow-up	63.36 ± 6.30	66.67 ± 6.17	0.113
Interval change			
Pre-AVR – post-AVR	–2.08 ± 8.21	–2.38 ± 9.47	0.922
Pre-AVR – follow-up	1.42 ± 6.95	3.52 ± 8.83	0.322

Values are presented as mean ± standard deviation

AVR, aortic valve replacement; EF, ejection fraction; GLS, global longitudinal strain; IVSd, interventricular septal dimension; LVMI, left ventricular mass index; RWT, relative wall thickness

^*^P < 0.05

A significant GLS-by-time interaction (interval changes) was observed for LVMI, at both the early post-SAVR phase (P = 0.030) and follow-up (P = 0.005), indicating differential regression of LVM according to baseline GLS status. However, no significant interaction was identified for other remodeling parameters, including IVSd, RWT, and LVEF at either phase (all P > 0.05). These findings suggest that baseline LV GLS may be associated with selected aspects of reverse remodeling, particularly LV mass regression, but may not consistently predict the overall magnitude of post-SAVR reverse remodeling.

These complete-case findings were materially consistent with those obtained using the full cohort of 94 patients (Table S2), in which the LVMI interaction at follow-up remained significant (P = 0.005), whereas IVSd, RWT, and EF interactions remained nonsignificant.

The complete-case and full-cohort analyses indicate that baseline LV GLS may be associated with selected aspects of reverse remodeling, particularly LVM regression, but does not consistently predict the overall magnitude of structural and functional remodeling after SAVR.

### Continuous LV GLS and magnitude of remodeling

To further investigate the relationship between preoperative GLS and the magnitude of reverse remodeling, we performed a continuous-variable analysis as a supplement to the dichotomous group comparison (Tables S3, S4).

Baseline LV GLS (as a continuous variable) was significantly associated with absolute LV indices after AVR and at follow-up, reflecting persistent baseline disease burden rather than a predictive relationship with postoperative change. Critically, baseline LV GLS showed no significant association with the magnitude of Δ in IVSd, LVMI, or RWT at either the early post-AVR phase (all P > 0.05) or follow-up (ΔIVSd, P = 0.264; ΔLVMI, P = 0.083; ΔRWT, P = 0.711). The significant correlation between baseline LV GLS and ΔLVEF at follow-up (ρ = + 0.43, P = 0.001) indicates that patients with worse GLS (less negative values) experienced greater EF recovery consistent with regression toward the mean and the law of initial values, not a limitation of remodeling.

### Multivariable regression and adjustment for baseline disease severity

Because the two GLS groups were unbalanced (normal GLS vs. abnormal GLS: full cohort, n = 22 vs. n = 72; complete-case cohort, n = 12 vs. n = 42) and differed significantly at baseline in sex, peak and mean transvalvular gradient, and AVA, we performed a post hoc power analysis (Table 6) and multivariable regression adjusting for these baseline differences (Table 7).

**Table 6:** Power analysis for the GLS × time interaction

Outcome	No. of patients		Cohen d	Power (%)
	Normal GLS	Abnormal GLS		
Phase 1 (full cohort)	22	72		
ΔIVSd			0.106	7.2
ΔLVMI			0.392	36.3
ΔEF			0.000	5.0
Phase 2 (complete-case cohort)	12	42		
ΔIVSd			0.345	18.4
ΔLVMI			0.777	66.0
ΔEF			0.249	11.8

Phase 1 (SAVR-to-post-SAVR echocardiography interval) had minimum Cohen d of 0.69 for 80% power. Phase 2 (SAVR-to-follow-up echocardiography interval) had minimum Cohen d of 0.92 for 80% power

EF, ejection fraction; GLS, global longitudinal strain; IVSd, interventricular septal dimension; LVMI, left ventricular mass index; RWT, relative wall thickness; SAVR, surgical aortic valve replacement

**Table 7:** Multivariable regression of Δ remodeling outcomes on continuous baseline GLS

Outcome (Δ)	Baseline GLS		Model 1			Model 2		
	β	P-value	β	*P*-value	R^2^	β	*P*-value	R^2^
Phase 1 (n = 94)								
ΔIVSd	0.294	0.181	0.174	0.502	0.238	0.317	0.322	0.270
ΔLVMI	6.230	0.080	3.220	0.404	0.425	0.320	0.944,	0.439
ΔEF	− 0.790	0.502	− 2.800	0.048^*^	0.332	− 2.440	0.153	0.343
Phase 2 (n = 54)								
ΔIVSd	0.646	0.016^*^	0.601	0.054	0.497	0.632	0.121	0.528
ΔLVMI	8.410	0.047^*^	9.580	0.058	0.661	7.680	0.214	0.679
ΔEF	− 0.080	0.942	− 0.600	0.646	0.473	1.460	0.343	0.548

Phase 1 (SAVR-to-post-SAVR echocardiography interval), baseline vs. post-SAVR echocardiography. Phase 2 (SAVR-to-follow-up echocardiography interval), baseline vs. follow-up echocardiography. Model 1 was adjusted for the baseline value of the respective outcome, peak transvalvular gradient, mean transvalvular gradient, and aortic valve area. Model 2 was additionally adjusted for sex, smoking status, and tricuspid annular plane systolic excursion

EF, ejection fraction; GLS, global longitudinal strain; IVSd, interventricular septal dimension; LVMI, left ventricular mass index; SAVR, surgical aortic valve replacement

^*^P < 0.05

With normal GLS (n = 12) and abnormal GLS (n = 42) in phase 2, the study had 80% power only to detect effect sizes of Cohen d ≥ 0.92, a threshold substantially larger than the observed effects for ΔIVSd (d = 0.35, power 18%) and ΔEF (d = 0.25, power 12%). This limitation is acknowledged explicitly. The group imbalance reflects the real-world prevalence of impaired GLS among patients with severe AS referred for SAVR rather than a sampling artifact. Phase 1, which used the complete cohort of 94 patients with substantially greater power, yielded entirely consistent null findings across all structural parameters, providing independent corroboration of the phase 2 results.

Furthermore, the observed effect sizes for ΔIVSd, ΔRWT, and ΔEF were small in both phases (all d < 0.35), falling well below any threshold of clinical relevance; this combination of nonsignificant results and small effect sizes supports the absence of a biologically meaningful relationship, rather than reflecting insufficient power to detect a true effect.

We performed multivariable regression adjusting for all baseline variables that differed significantly between GLS groups and were available in the dataset: the baseline value of each outcome parameter, peak transvalvular gradient, mean transvalvular gradient, AVA, sex, smoking status, and TAPSE. In this adjusted model, continuous baseline LV GLS was not independently associated with any of the six remodeling outcomes (all P ≥ 0.121). Notably, the borderline associations observed in the unadjusted model for phase 2 ΔIVSd (P = 0.016) and ΔLVMI (*P* = 0.047) became nonsignificant after adjustment (*P* = 0.121 and P = 0.214, respectively), indicating that these apparent associations were attributable to confounding by baseline disease severity, as reflected by transvalvular gradients, AVA, and baseline structural indices, rather than an independent effect of LV GLS.

### Secondary analysis: LMM analysis

As a secondary, exploratory analysis relying on the MAR assumption, an LMM using all available data (n = 94) was fitted for each outcome. The LMM, which included all available data, revealed significant main effects for both time and GLS category, but no significant interaction between them (Akaike information criterion: main effects model, 834.5 vs. interaction model, 837.9). The detailed parameters of the final model are listed in Table 8.

**Table 8:** Linear mixed-effects model parameters for reverse remodeling

Parameter	Coefficient (β)	95% CI	*P*-value
IVSd			
GLS category (normal vs. abnormal)	–2.33	–3.71 to –0.96	0.001^*^
Time (follow-up vs. pre-AVR)	–2.26	–2.95 to –1.58	< 0.001^*^
GLS × time interaction	-	-	0.511
LVMI			
GLS category (normal vs. abnormal)	–51.00	–73.54 to –28.46	< 0.001^*^
Time (follow-up vs. pre-AVR)	–57.78	–70.19 to –45.38	< 0.001^*^
GLS × time interaction	-	-	0.081
RWT			
GLS category (normal vs. abnormal)	–0.06	–0.13 to 0.01	0.109
Time (follow-up vs. pre-AVR)	–0.04	–0.09 to 0.01	0.134
GLS × time interaction	-	-	0.278
EF			
GLS category (normal vs. abnormal)	4.50	0.480 to 8.52	0.028^*^
Time (follow-up vs. pre-AVR)	3.27	0.35 to 6.20	0.028^*^
GLS × time interaction	-	-	0.809

Model: IVSd ~ GLS_category + Time + (1|Subject_ID). Akaike information criterion** = **834.5

AVR, aortic valve replacement; CI, confidence interval; EF, ejection fraction; GLS, global longitudinal strain; IVSd, interventricular septal dimension; LVMI, left ventricular mass index; RWT, relative wall thickness

^*^P < 0.05

**Fig. 1:**
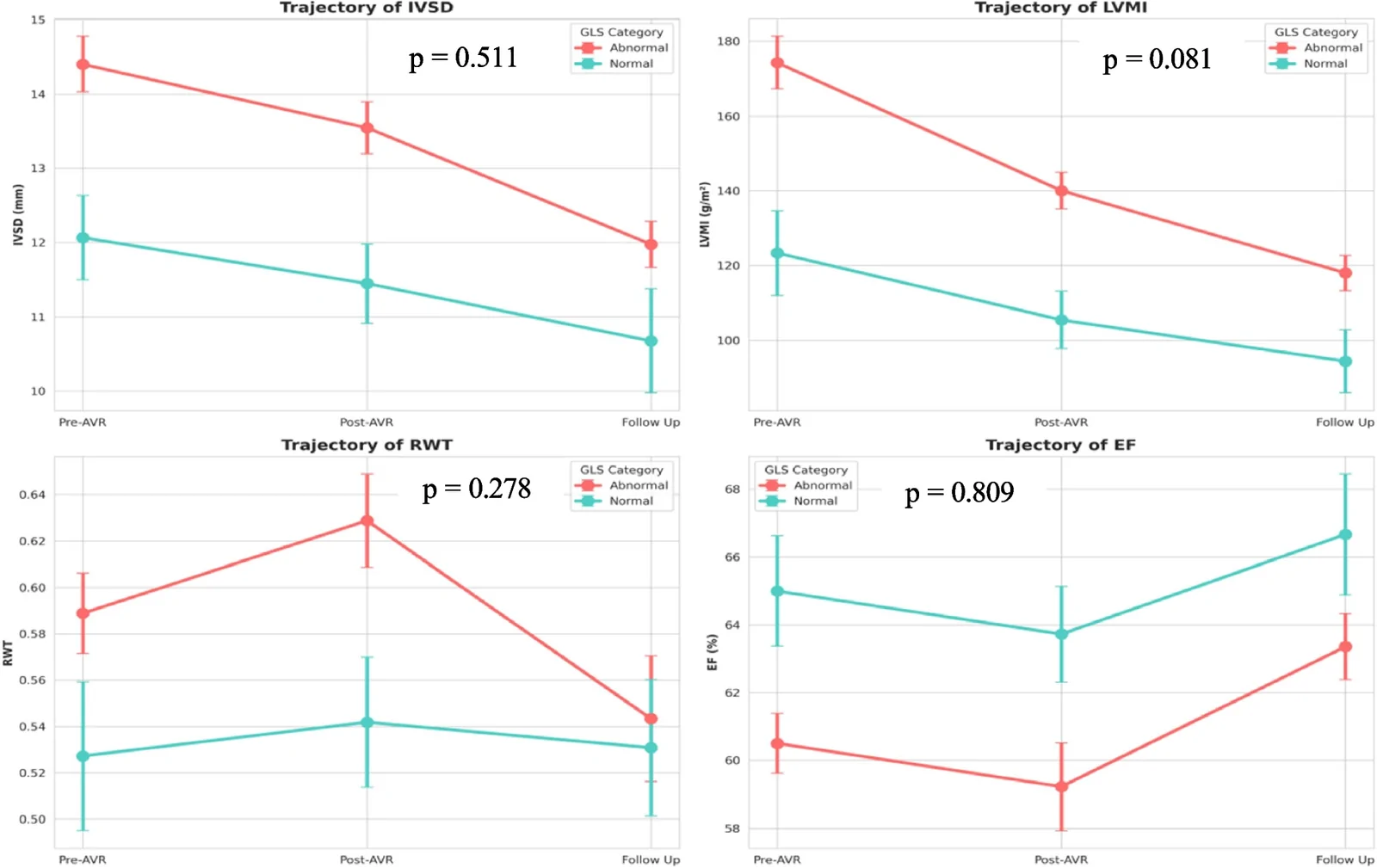
Trajectory plots showing longitudinal trends in mean **A** interventricular septal dimension (IVSd), **B** left ventricular mass index (LVMI), **C** relative wall thickness (RWT), and **D** ejection fraction (EF) values across the study time points. AVR, aortic valve replacement; GLS, global longitudinal strain

We analyzed reverse-remodeling echocardiographic parameters in the full cohort, including IVSd, LVMI, RWT, and EF. The results of the LMM analyses are summarized in Table 8. The secondary analysis is presented graphically in Fig. 1, illustrating the longitudinal trends in mean IVSd, LVMI, RWT, and EF across the study time points for each GLS group.

#### Interventricular septal dimension

A significant main effect was found for GLS category (β** = **–2.33; 95% confidence interval [CI], –3.71 to –0.96; P** = **0.001), indicating that the normal GLS group had, on average, an IVSd value 2.33 mm lower than the abnormal GLS group after accounting for time. There was also a highly significant main effect of time (β** = **–2.26; follow-up vs. baseline, P < 0.001), reflecting substantial septal thinning following SAVR.

#### Left ventricular mass index

Significant main effects were found for both GLS category (β** = **–51.00, P < 0.001) and time (P < 0.001), with no significant interaction (P** = **0.081). This indicates that while the abnormal GLS group had a much higher baseline LVMI, both groups experienced a similar and substantial magnitude of mass regression.

#### Relative wall thickness

A nonsignificant main effect was observed for GLS category (β** = **–0.06, P** = **0.109), and there was no significant change over time (P** = **0.134), suggesting that the concentric remodeling pattern remained stable in the early post-SAVR period.

#### Ejection fraction

The normal GLS group had a significantly higher EF at baseline (β** = **4.50, P** = **0.028). A significant improvement in EF was observed only at follow-up (β** = **3.27; vs. baseline, P** = **0.028), with no significant interaction effect.

### Sensitivity analysis

To assess the robustness of the results in relation to missing data, the LMM analysis was repeated in a subset of 54 patients with complete data at all three time points. The results were highly consistent with the primary analysis, yielding similar effect sizes and levels of statistical significance for both the GLS and time effects. This consistency confirms that the missing data did not introduce substantial bias into the model's estimates.

## Discussion

This study examined LV reverse remodeling after SAVR in patients with severe AS and preserved EF, using a complete-case analysis and continuous-variable correlation as the principal analytic strategy, supplemented by a secondary LMM. Across all three approaches, preoperative GLS correlated with the severity of myocardial hypertrophy but, with the exception of LVM, did not independently predict the magnitude of postoperative LV reverse remodeling once baseline disease severity was accounted for. This finding challenges the assumption that impaired subclinical function limits the heart's restorative capacity and provides a more nuanced understanding of the determinants of postprocedural recovery.

Previous studies have suggested that GLS detects subclinical LV dysfunction in patients with severe AS, determines LV functional recovery after aortic valve surgery, and predicts mortality independent of other known predictors. In this study, several important findings emerged. First, abnormal baseline GLS (≥ –16%) was associated with a distinctly more advanced myocardial phenotype, including greater septal thickness, higher LVMI and RWT, and reduced EF, consistent with GLS reflecting baseline disease burden [6, 13]. Second, we identified a clear and progressive pattern of reverse remodeling, with sustained reductions in LVMI both early after SAVR and at follow-up in the abnormal GLS group, suggesting that baseline GLS status may be associated with the degree of LVM regression specifically, even though continuous GLS was not independently associated with ΔLVMI after adjustment for baseline disease severity. Third, the magnitude of this remodeling, tested directly via the GLS × time interaction in the complete-case and full-cohort comparisons, via continuous-variable correlation with Δ outcomes, and via multivariable regression adjusting for baseline disease severity, was statistically indistinguishable between GLS groups for IVSd, RWT, and EF.

Although unexpected at first glance, our results fit within the evolving understanding of GLS serving two separate functions in AS: as a marker of baseline myocardial integrity (risk prediction) and as a potential determinant of postoperative improvement (benefit prediction). The present data support the view that GLS is primarily a risk indicator rather than a determinant of postoperative benefit. This interpretation is consistent with recent evidence: Skulstad et al. found that only baseline LVMI and hypertension predicted regression in LVMI after transcatheter aortic valve implantation, with baseline GLS showing no predictive contribution [9].

Similarly, Al-Rashid et al. [7] reported that while GLS reliably predicted adverse events, supporting its role as a marker of myocardial disease burden, it did not correlate with the magnitude of functional or structural improvement. These findings collectively underscore that impaired GLS signifies disease burden but does not limit the capacity for reverse remodeling.

The observed dissociation is consistent with Wilder principle, the law of initial values, whereby the initial burden of disease largely dictates the magnitude of improvement. Patients with abnormal GLS who demonstrated more pronounced hypertrophy at baseline inherently had a larger window for regression. Their ability to achieve reductions similar to those with normal GLS suggests that impaired preoperative GLS does not diminish the remodeling potential. Bennett et al. [3] provide complementary mechanistic data, showing that higher baseline myocyte volume is the dominant predictor of myocyte regression after AVR. The significant GLS × time interaction for LVMI in our complete-case analysis, together with the attenuation of the unadjusted GLS – ΔLVMI association after adjustment for baseline severity, suggests that LVM regression specifically may be more closely tied to the combination of baseline mass and subclinical dysfunction than the other structural parameters examined here; this distinction merits further confirmation in larger, multicenter cohorts.

Therefore, we propose a conceptual model where GLS and baseline structure play distinct roles. Baseline GLS is an indicator of myocardial health and clinical risk. An abnormal GLS reflects myocardial damage and is associated with underlying fibrotic remodeling, which predisposes patients to adverse clinical events such as heart failure and mortality, as has been extensively documented [7, 8, 14, 15]. Baseline structure (IVSd and LVMI) acts as an indicator of remodeling severity and potential for recovery. The degree of hypertrophy is the primary determinant of the magnitude of structural regression once the pathological stimulus (pressure overload) is removed.

The findings of this study have significant clinical implications. They suggest that the presence of subclinical myocardial dysfunction, as indicated by an abnormal GLS, should not be interpreted as a sign of irreversible structural impairment or limited capacity for recovery. Clinicians should not be discouraged from recommending AVR for patients with impaired GLS, as these patients stand to gain a comparable, if not greater, degree of beneficial structural remodeling. Our results advocate for a shift in the clinical application of GLS in this population. Rather than using it to predict the benefit of AVR (i.e., the degree of reverse remodeling), GLS should primarily be used to predict the risk associated with the procedure and the patient's long-term prognosis. Patients with severe AS and abnormal GLS are at high risk and may require more intensive perioperative monitoring and aggressive postoperative management; however, they are also patients who are poised to achieve significant structural improvement.

Cardiac magnetic resonance (CMR) with late gadolinium enhancement (LGE) and T1 mapping are the gold standards for assessing focal and diffuse fibrosis, respectively [16, 17]. Evidence shows that myocardial fibrosis is a critical determinant of AS outcomes. The presence of LGE, indicating replacement fibrosis, is a powerful predictor of all-cause mortality and a powerful prognostic marker [18, 19], with a meta-analysis by Papanastasiou et al. [20] showing that it confers a more than twofold higher risk.

GLS has been shown to correlate closely with myocardial fibrosis and LV remodeling; therefore, it serves as a functional marker of the underlying myocardial disease burden [21]. Impairment in longitudinal strain is thought to occur because the subendocardial layer is particularly vulnerable to ischemia and fibrosis. Future studies should aim to integrate GLS with CMR fibrosis quantification to build more comprehensive and accurate predictive models. It is plausible that patients with abnormal GLS but without significant LGE have the greatest potential for both structural and functional recovery, whereas those with both abnormal GLS and extensive LGE represent a high-risk group with a more limited ceiling for improvement.

Our findings are broadly consistent with contemporary trials evaluating earlier intervention in asymptomatic severe AS, such as EARLY TAVR and EVOLVED trials, which addressed the distinct questions of early versus delayed intervention timing and fibrosis-guided patient selection, respectively [22–24]. We do not claim that our data provide a mechanistic basis for these trials; rather, our observation that even patients with subclinical dysfunction (abnormal GLS) retain the capacity for substantial early structural recovery is consistent with and supportive of the broader rationale for not delaying intervention in this population. The decision of when to intervene, however, depends on a range of factors beyond remodeling capacity, including symptom status, fibrosis burden, and procedural risk, which were not the focus of this study.

These considerations reinforce the role of GLS primarily as a risk stratification tool. These findings are closely aligned with the 2025 scientific statement from the American Heart Association emphasizing the prognostic value of GLS, which notes that impaired GLS > − 16% in patients with moderate AS and preserved LVEF has been associated with a twofold increase in mortality risk [5], similar to that observed in patients with reduced LVEF [11]. Our findings complement this prognostic role by clarifying that, with the possible exception of LVM, an abnormal GLS does not itself signal a diminished capacity for structural recovery after SAVR; rather, it should prompt closer risk stratification and shared decision-making about the timing of intervention, independent of any expectation regarding the magnitude of postprocedural remodeling.

### Limitations

The primary strength of our study is its longitudinal design with three measurement points, analyzed principally via a complete-case approach supplemented by continuous-variable correlation, multivariable adjustment, and a secondary mixed-effects model, thereby providing convergent evidence from complementary analytic strategies.

Several limitations of this study must be acknowledged. First, this was a retrospective, single-center study, which may limit the generalizability of our findings. Second, follow-up was limited and varied in duration (8.5** ± **6.2 months); therefore, the long-term trajectories of reverse remodeling may differ. Third, we did not have data on myocardial fibrosis from CMR, which would have provided a more direct measure of irreversible myocardial damage. The interplay between diffuse interstitial fibrosis (reflected by GLS) and focal scars (detected by LGE) in modulating reverse remodeling warrants further investigation [25, 26]. Fourth, serial post-AVR LVIDd, LVEDV, and LV GLS data were incomplete and were therefore excluded from the continuous-variable analyses to avoid introducing additional bias. Finally, the sample size, particularly in the normal GLS group, was relatively small, which could limit the power to detect a small interaction effect.

## Conclusions

Our study demonstrates that in patients with severe AS and preserved EF, baseline GLS is a powerful marker of the severity of existing LV hypertrophy but does not predict the magnitude of early structural reverse remodeling following SAVR. A complete-case primary analysis, continuous-variable correlation, and adjustment for baseline disease severity showed that baseline GLS did not independently predict the overall magnitude of early structural reverse remodeling after SAVR, with the possible exception of a modest association with LVM regression. The capacity for beneficial structural remodeling in IVSd, RWT, and EF appears to be preserved even in the presence of significant subclinical myocardial dysfunction. This finding provides a reassuring message for clinicians and patients, supporting the continued application of AVR across a broad spectrum of patients with severe AS and clarifying the role of GLS as a critical tool for risk stratification rather than a predictor of structural recovery potential.

## Supplementary Information

Additional file 1: Table S1. Direct comparison of echocardiographic timing between GLS groups. Table S2. Comparison of reverse remodeling parameters by GLS group, full cohort (n=94). Table S3. Spearman correlations: baseline LV GLS (Continuous) vs. absolute LV remodeling indices. Table S4. Spearman correlations: baseline LVGLS (Continuous) vs. Δ LV remodeling indices

## Data Availability

The authors confirm that the data supporting the findings of this study are available within the article.

## Abbreviations

AS: Aortic stenosis
ASE: American Society of Echocardiography
AVA: Aortic valve area
AVR: Aortic valve replacement
CI: Confidence interval
CMR: Cardiac magnetic resonance
EF: Ejection fraction
GLS: Global longitudinal strain
IVSd: Interventricular septal dimension
LGE: Late gadolinium enhancement
LMM: Linear mixed-effects model
LV: Left ventricular
LVEDV: Left ventricular end-diastolic volume
LVEF: Left ventricular ejection fraction
LVESV: Left ventricular end-systolic volume
LVIDd: Left ventricular internal dimension diastole
LVM: Left ventricular mass
LVMI: Left ventricular mass index
MAR: Missing at random
NCCHK: National Cardiovascular Center Harapan Kita
PWd: Posterior wall thickness
RWT: Relative wall thickness
SAVR: Surgical aortic valve replacement
TAPSE: Tricuspid annular plane systolic excursion
