# Correction: Preoperative global longitudinal strain predicts myocardial hypertrophy burden but not the magnitude of reverse remodeling after surgical aortic valve replacement in severe aortic stenosis

**DOI:** 10.1186/s44348-026-00102-8

**Published:** 2026-10-10

**Authors:** Joy Wulansari Purba, Ario Soeryo Kuncoro, Rina Ariani, Estu Rudiktyo, Amiliana Mardiani Soesanto

**Affiliations:** 1 Department of Cardiovascular, Adam Malik Hospital, Medan, Indonesia; 2 Department of Cardiology and Vascular Disease, Faculty of Medicine, Universitas Sumatera Utara, Medan, Indonesia; 3 Department of Cardiology and Vascular Medicine, Faculty of Medicine, Universitas Indonesia. National Cardiovascular Center Harapan Kita, Jakarta, Indonesia

**Correction: J Cardiovasc Imaging 34, 30 (2026)**

**https://doi.org/10.1186/s44348-026–00089-2**

In the original publication of this article [1], the time-point values (Pre-AVR, Post-AVR and Follow-up) in Table 5 and in Supplementary Table S2 were presented under the wrong GLS group headings ("Normal GLS" and "Abnormal GLS"). The "Interval changes" rows were correctly assigned and are unchanged. The corrected tables are shown below. The Results text, Discussion and Conclusions describe the two groups correctly and are not affected.

**1. Corrected Table 5**

**Complete-case comparison of reverse remodeling parameters by GLS group (** ***n*** **= 54)**

**Table 5:** Complete-case comparison of reverse remodeling parameters by GLS group (*n* = 54)

Parameter	Normal GLS (n=12)	Abnormal GLS (n=42)	*P*-value
**IVSd (mm)**			
Pre AVR	11.94 ± 2.50	14.14 ± 2.51	0.010
Post AVR	11.28 ± 2.12	13.39 ± 2.56	0.012
Follow up	10.68 ± 2.42	11.97 ± 2.02	0.053
**Interval changes**			
Pre AVR – Post AVR	−0.66 ± 1.79	−0.74 ± 2.33	0.787
Pre AVR – Follow up	−1.27 ± 1.21	−2.16 ± 2.86	0.208
**LVMI (g/m**^**2**^)			
Pre AVR	110.20 ± 29.80	177.30 ± 65.00	< 0.001
Post AVR	100.50 ± 24.10	140.00 ± 35.70	< 0.001
Follow up	94.40 ± 29.20	118.00 ± 30.60	0.015
**Interval changes**			
Pre AVR – Post AVR	−9.80 ± 37.30	−37.30 ± 51.00	0.030
Pre AVR – Follow up	−15.80 ± 32.20	−59.30 ± 60.90	0.005
**RWT**			
Pre AVR	0.52 ± 0.14	0.56 ± 0.11	0.212
Post AVR	0.51 ± 0.12	0.62 ± 0.15	0.006
Follow up	0.53 ± 0.10	0.54 ± 0.17	>0.999
**Interval changes**			
Pre AVR – Post AVR	−0.01 ± 0.17	0.06 ± 0.17	0.246
Pre AVR – Follow up	0.01 ± 0.18	−0.02 ± 0.21	0.588
**EF (%)**			
Pre AVR	65.25 ± 5.71	59.83 ± 7.13	0.013
Post AVR	63.17 ± 5.06	57.45 ± 8.78	0.036
Follow up	66.67 ± 6.17	63.36 ± 6.30	0.113
**Interval changes**			
Pre AVR – Post AVR	−2.08 ± 8.21	−2.38 ± 9.47	0.922
Pre AVR – Follow up	1.42 ± 6.95	3.52 ± 8.83	0.322

**2. Corrected Supplementary Table S2 (** ***n*** **= 94)**

**Comparison of reverse remodeling parameters by GLS group, full cohort (*n* = 94)**

**Table:** 

Parameter	Normal GLS	Abnormal GLS	*p*-value
**IVSd (mm)**			
Pre-AVR	12.06 ± 2.67	14.40 ± 3.16	0.002
Post-AVR	11.45 ± 2.50	13.55 ± 2.98	0.002
Follow-up	10.68 ± 2.42	11.97 ± 2.02	0.042
**Interval changes**			
Δ Pre-AVR → Post-AVR	−0.62 ± 1.44	−0.86 ± 2.42	0.961
Δ Pre-AVR → Follow-up	−1.27 ± 1.21	−2.16 ± 2.86	0.208
**LVMI (g/m**^**2**^**)**			
Pre-AVR	123.31 ± 53.46	174.30 ± 59.30	< 0.001
Post-AVR	105.47 ± 35.84	140.00 ± 41.50	< 0.001
Follow-up	94.42 ± 29.20	118.00 ± 30.60	0.013
**Interval changes**			
Δ Pre-AVR → Post-AVR	−17.80 ± 39.80	−34.90 ± 44.50	0.087
Δ Pre-AVR → Follow-up	−15.80 ± 32.20	−59.30 ± 60.90	0.005
**RWT**			
Pre-AVR	0.53 ± 0.15	0.59 ± 0.15	0.091
Post-AVR	0.54 ± 0.13	0.63 ± 0.17	0.025
Follow-up	0.53 ± 0.10	0.54 ± 0.18	0.789
**Interval changes**			
Δ Pre-AVR → Post-AVR	−0.01 ± 0.15	0.04 ± 0.19	0.563
Δ Pre-AVR → Follow-up	0.01 ± 0.18	−0.02 ± 0.21	0.588
**LVEF (%)**			
Pre-AVR	65.00 ± 7.61	60.50 ± 7.48	0.016
Post-AVR	63.73 ± 6.63	59.22 ± 10.92	0.098
Follow-up	66.67 ± 6.17	63.36 ± 6.30	0.218
**Interval changes**			
Δ Pre-AVR → Post-AVR	−1.27 ± 11.48	−1.28 ± 11.30	0.999
Δ Pre-AVR → Follow-up	1.42 ± 6.95	3.52 ± 8.83	0.322
