# Supplementary material for: Preoperative global longitudinal strain predicts myocardial hypertrophy burden but not the magnitude of reverse remodeling after surgical aortic valve replacement in severe aortic stenosis
Source: J Cardiovasc Imaging. 2026 Sep 4;34:30. doi: 10.1186/s44348-026-00089-2 (PMC13543429; doi:10.1186/s44348-026-00089-2)
Supplement: Supplementary file 1 [file 44348_2026_89_MOESM1_ESM.docx]

**SUPPLEMENTARY MATERIAL**

*Preoperative Global Longitudinal Strain Predicts Myocardial Hypertrophy Burden But Not The Magnitude of Reverse Remodeling After Surgical Aortic Valve Replacement in Severe Aortic Stenosis*

# **Supplementary Table S1**

**Direct Comparison of Echocardiographic Timing Between GLS Groups**

| **Time interval** | **Normal GLS, n** | **Normal GLS mean ± SD (days)** | **Abnormal GLS, n** | **Abnormal GLS mean ± SD (days)** | **p-value** |
| --- | --- | --- | --- | --- | --- |
| Baseline to SAVR | 22 | 61.32 ± 40.71 | 72 | 81.93 ± 83.95 | 0.122 |
| SAVR to Post-SAVR | 22 | 6.0 ± 5.4 | 72 | 7.2 ± 6.1 | 0.617 |
| Baseline to Follow-up | 12 | 323.33 ± 195.12 | 42 | 337.86 ± 227.35 | 0.829 |
| SAVR to Follow-up | 12 | 268.33 ± 183.27 | 42 | 251.48 ± 190.47 | 0.784 |

# *Both timing intervals are non-normally distributed (Shapiro–Wilk p<0.001); p-values were calculated using a two-sided Welch independent t-test. Intervals involving follow-up only include subjects with available follow-up dates; the number of valid data points for the SAVR-to-follow-up and baseline-to-follow-up intervals is therefore smaller than for the baseline-to-SAVR interval.*

# **Supplementary Table S2**

**Comparison of Reverse Remodeling Parameters by GLS Group, Full Cohort (n=94)**

| **Parameter** | **Normal GLS** | **Abnormal GLS** | **p-value** |
| --- | --- | --- | --- |
| **IVSd (mm)** |  |  |  |
| Pre-AVR | 14.40 ± 3.16 | 12.06 ± 2.67 | 0.002 |
| Post-AVR | 13.55 ± 2.98 | 11.45 ± 2.50 | 0.002 |
| Follow-up | 11.97 ± 2.02 | 10.68 ± 2.42 | 0.042 |
| **Interval changes** | | | |
| Δ Pre-AVR→Post-AVR | -0.62 ± 1.44 | -0.86 ± 2.42 | 0.961 |
| Δ Pre-AVR→Follow-up | -1.27 ± 1.21 | -2.16 ± 2.86 | 0.208 |
| **LVMI (g/m²)** |  |  |  |
| Pre-AVR | 174.30 ± 59.30 | 123.31 ± 53.46 | <0.001 |
| Post-AVR | 140.00 ± 41.50 | 105.47 ± 35.84 | <0.001 |
| Follow-up | 118.00 ± 30.60 | 94.42 ± 29.20 | 0.013 |
| **Interval changes** |  |  |  |
| Δ Pre-AVR→Post-AVR | -17.80 ± 39.80 | -34.90 ± 44.50 | 0.087 |
| Δ Pre-AVR→Follow-up | -15.80 ± 32.20 | -59.30 ± 60.90 | 0.005 |
| **RWT** |  |  |  |
| Pre-AVR | 0.59 ± 0.15 | 0.53 ± 0.15 | 0.091 |
| Post-AVR | 0.63 ± 0.17 | 0.54 ± 0.13 | 0.025 |
| Follow-up | 0.54 ± 0.18 | 0.53 ± 0.10 | 0.789 |
| **Interval changes** |  |  |  |
| Δ Pre-AVR→Post-AVR | -0.01 ± 0.15 | 0.04 ± 0.19 | 0.563 |
| Δ Pre-AVR→Follow-up | 0.01 ± 0.18 | -0.02 ± 0.21 | 0.588 |
| **LVEF (%)** |  |  |  |
| Pre-AVR | 60.50 ± 7.48 | 65.00 ± 7.61 | 0.016 |
| Post-AVR | 59.22 ± 10.92 | 63.73 ± 6.63 | 0.098 |
| Follow-up | 63.36 ± 6.30 | 66.67 ± 6.17 | 0.218 |
| **Interval changes** | | | |
| Δ Pre-AVR→Post-AVR | -1.27 ± 11.48 | -1.28 ± 11.30 | 0.999 |
| Δ Pre-AVR→Follow-up | 1.42 ± 6.95 | 3.52 ± 8.83 | 0.322 |

# **Supplementary Table S3**

**Spearman Correlations — Baseline LVGLS (Continuous) vs. Absolute LV Remodeling Indices**

| **Outcome** | **Baseline rs (n=94)** | **p-value** | **Post-AVR rs (n=94)** | **p-value** | **Follow-up rs (n=54)** | **p-value** |
| --- | --- | --- | --- | --- | --- | --- |
| IVSd | 0.251 | 0.015 | 0.306 | 0.003 | 0.393 | 0.003 |
| LVMI | 0.510 | <0.001 | 0.436 | <0.001 | 0.382 | 0.004 |
| RWT | 0.079 | 0.448 | 0.229 | 0.026 | 0.057 | 0.683 |
| LVEF | -0.463 | <0.001 | -0.204 | 0.048 | -0.150 | 0.278 |

# **Supplementary Table S4**

**Spearman Correlations — Baseline LVGLS (Continuous) vs. Δ LV Remodeling Indices**

| **Outcome** | **Δ Post-AVR rs (n=94)** | **p-value** | **Δ Follow-up rs (n=54)** | **p-value** |
| --- | --- | --- | --- | --- |
| IVSd | 0.073 | 0.486 | 0.155 | 0.264 |
| LVMI | -0.199 | 0.056 | -0.238 | 0.083 |
| RWT | 0.113 | 0.280 | 0.052 | 0.711 |
| LVEF | 0.189 | 0.068 | 0.43 | 0.001 |
